# Antimicrobial and Cytotoxic Isohexenylnaphthazarins from *Arnebia euchroma *(Royle) Jonst. (Boraginaceae) Callus and Cell Suspension Culture

**DOI:** 10.3390/molecules171214310

**Published:** 2012-12-03

**Authors:** Harilaos Damianakos, Nadine Kretschmer, Katarzyna Sykłowska-Baranek, Agnieszka Pietrosiuk, Rudolf Bauer, Ioanna Chinou

**Affiliations:** 1 Department of Pharmacy, Institute of Pharmacognosy & Chemistry of Natural Products, University of Athens, Zografou 15771, Athens, Greece; E-Mail: harisdam@pharm.uoa.gr; 2 Department of Pharmacognosy, Institute of Pharmaceutical Sciences, Karl-Franzens University, Universitätsplatz 4, 8010 Graz, Austria; E-Mails: nadine.kretschmer@uni-graz.at (N.K.); rudolf.bauer@uni-graz.at (R.B.); 3 Department of Biology and Pharmaceutical Botany, Medical University of Warsaw, Banacha Street 1, 02-097 Warsaw, Poland; E-Mails: kasiasb@farm.amwaw.edu.pl (K.S.-B.); apietrosiuk@yahoo.co.uk (A.P.)

**Keywords:** *Arnebia euchroma*, Boraginaceae, callus culture, cell suspension culture, isohexenylnaphthazarins, cytotoxic, antimicrobial

## Abstract

The phytochemical investigation of the *n*-hexane extract from callus and cell suspension culture of *Arnebia euchroma* (Royle) Jonst. resulted in the isolation of nine isohexenylnaphthazarins: deoxyalkannin (**1**), alkannin (**2**), acetylalkannin (**3**), isobutyrylalkannin (**4**), β*-*hydroxyisovalerylalkannin (**5**), 2''-(*S*)-α-methylbutyrylalkannin (**6**), propionylalkannin (**7**), teracrylalkannin (**8**) and acetylshikonin (**9**). Their structures were determined by MS and NMR spectroscopy. Pigments **2**–**8** are isolated for the first time from *Arnebia in vitro* cultures, **4** and **7** are reported in the present work as novel metabolites within the *Arnebia* genus, while **9** is a known constituent of both natural roots and *in vitro* cultures of *A. euchroma.* Moreover, methyl jasmonate and 1-monoglyceryl olate, palmitate and stearate are reported for the first time within the Boraginaceae family. The antimicrobial and cytotoxic activities of all isolated pigment compounds were tested, revealing a very interesting profile.

## 1. Introduction

Alkannin, shikonin and related isohexenylnaphthazarin compounds are natural lipophilic red pigments that occur in many species of the Boraginaceae family. The chiral pairs of alkannins/shikonins are potent pharmaceutical substances that have shown significant biological activities including wound healing, antimicrobial, antiinflammatory, antioxidant, anticancer and antithrombotic properties [1,2,3,4]. Because of the importance of the alkannin/shikonin-related compounds in the pharmaceutical and cosmetic industry, cultivation of Boraginaceous species, including the genus *Arnebia*, have been studied using biotechnological approaches. *Arnebia euchroma* (Royle) Jonst. tissue culture was pioneered in Russia due to the plant’s high content of shikonin/alkannin derivatives [5]. In this study we were focused on the isolation of isohexenylnaphthazarin compounds from callus culture and cell suspension culture of *A. euchroma*, elucidation of their structures by modern spectral techniques and the evaluation of their antimicrobial and cytotoxic activities.

## 2. Results and Discussion

### 2.1. Isolation of Isohexenylnaphthazarin Pigments

**Figure 1:**
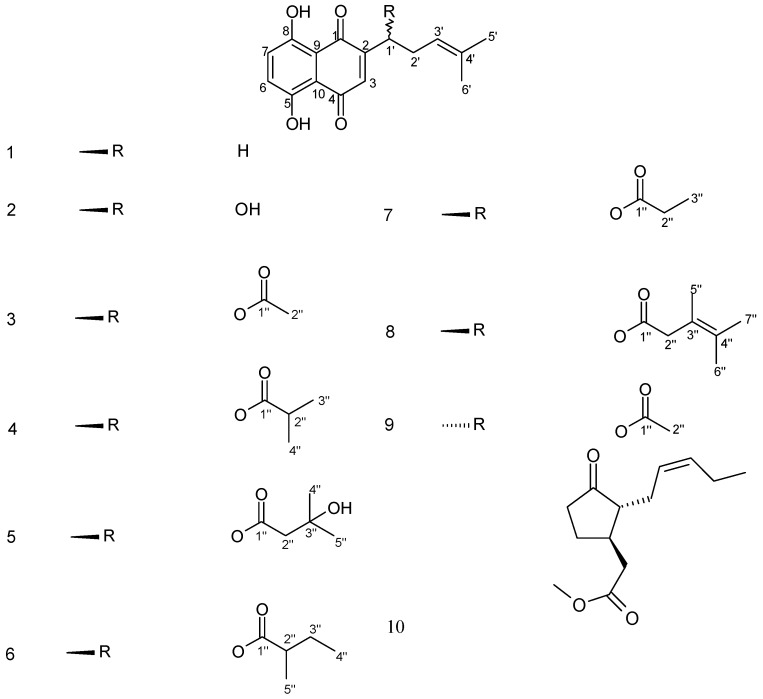
Chemical structures of compounds isolated from callus and cell suspension cultures of *A. euchroma*.

Isohexenylnaphthazarin pigments are well known constituents of certain plants of the Boraginaceae family [1,6,7,8,9,10,11,12,13,14,15]. The stereochemistry of the herein isolated isohexenylnaphthazarins was assigned to be of the alkannin type, except for acetylshikonin (Figure 1), on the basis of their negative specific rotation values [9,14], albeit in different solvents and at slightly different temperatures from those of the literature.

Regarding their natural abundance within the *Arnebia* genus, a review of the literature lead to the following findings: (a) compound **1** is abundant in the roots of various *Arnebia* species, including *A. euchroma*, and has also been isolated from cell cultures of the latter species [1]; (b) compound **2** is found in the roots of *A. hispidissima*, *A. nobilis*, *A. tinctoria* [1,15] and *A. euchroma* [13]; (c) compound **3** is found in the roots of *A. euchroma*, *A. hispidissima* and *A. nobilis* [1]; (d) compound **4** is not contained either in the natural roots or in any cell culture so far; (e) compound **5** occurs in the roots of *A. euchroma* and *A. hispidissima* [1]; (f) compound **6** occurs in the roots of *A. densiflora* [13,14,16] and *A. euchroma* [13]; (g) compound **7** is not produced in either natural roots or in *in vitro* cultures; (h) compound **8** is found in the roots of *A. densiflora* [15]; (i) compound **9** is a known constituent of both natural roots and *in vitro* cultures of *A. euchroma* [1].

Apart from naphthoquinone pigments, linoleic acid [17] and β-sitosterol [18] have been identified in both *in vitro* cultures. Additionally, methyl linolate [17] has been isolated from the cell suspension culture (also detected by GC-MS in the roots of other Boraginaceous species [19,20]) as well as methyl jasmonate (**10**) [21,22] (for the first time within the Boraginaceae family), a triglyceride mixture of palmitic, stearic and 8-*Ζ*- or 9-*Ζ*- or 11-*Ζ* or 9-*Ε*-octadecenoic acid (as determined after transesterification/methylation and GC-MS analysis of the resulting fatty acid methyl esters [23]) and also a mixture of 1-monoglycerides of palmitic, stearic and oleic acid (as determined after transesterification/methylation and GC-MS analysis of the resulting fatty acid methyl esters [23], as well as after GC-MS analysis of the silylated derivatives which were identified as 2,3-*O*-bis(trimethylsilyl)-1-glyceryl palmitate, stearate and oleate respectively) probably for the first time within Boraginaceae family.

### 2.2. Antimicrobial Activity

According to our results (Table 1) the *n*-hexane extract of both callus and cell suspension culture of *A. euchroma*, showed a very interesting broad antimicrobial profile against all the assayed microorganisms, while among the pure tested compounds the most active compounds were alkannin, acetylshikonin, β-hydroxyisovalerylalkannin and isobutyrylalkannin. It is noteworthy that acetylshikonin was much more active in comparison with its enantiomer acetylalkannin.

**Table 1:** Antimicrobial activities of extracts and isolated compounds (**1**–**9**) from *A. euchroma* (n = 3).

	Zone of inhibition in cm								
	*S. aureus*	*S. epidermidis*	*P. aeruginosa*	*K. pneumoniae*	*E. cloacae*	*E. coli*	*C. albicans*	*C. tropicalis*	*C. glabrata*
***A. euchroma *(**callus *n*-hexane extract)	15	15	12	11	11	11	10	11	12
***A. euchroma *(**cell suspension culture *n*-hexane extract)	16	16	13	12	12	12	12	13	13
**1**	12	12	11	10	11	12	9	10	10
**2**	15	14	12	11	11	12	11	12	14
**3**	10	11	10	10	10	9	9	10	10
**4**	14	13	13	11	11	12	10	12	13
**5**	15	15	13	13	12	14	11	12	12
**6**	13	12	12	11	11	12	10	11	12
**7**	12	13	11	12	12	11	10	11	11
**8**	12	11	12	10	10	11	10	11	11
**9**	15	14	12	12	11	11	12	11	13
**Metilmycin**	22	24	20	25	23	22	-	-	-
**Amoxicillin with clavulanic acid**	21	21	25	23	22	24	-	-	-
**5-Flucytocine**	-	-	-	-	-	-	22	24	22
**Amphotericin B**	-	-	-	-	-	-	23	24	24

### 2.3. Cytotoxic Activities

**Figure 2:**
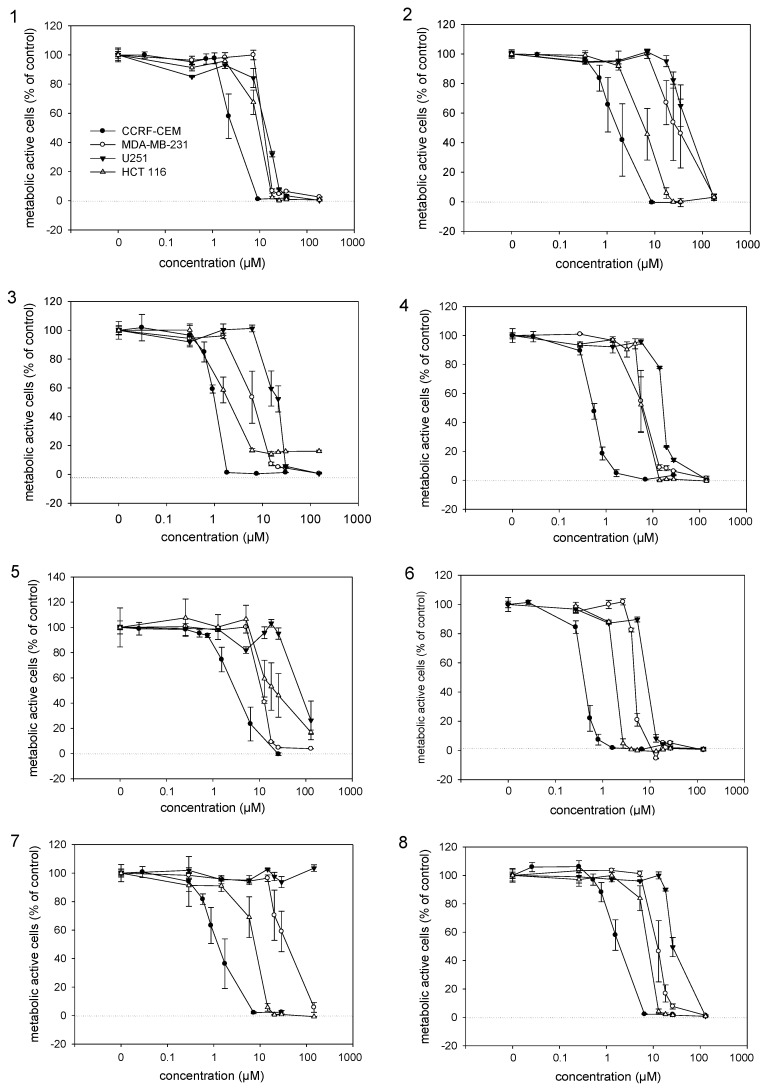
Cytotoxic activities of isolated alkannin derivatives determined by the XTT viability assay. Results represent mean ± sem, n = 4. Numbers correspond to the isolated compounds (**1**–**8**).

To study the cytotoxicity of the isolated naphthoquinone derivatives, they were subjected to a viability assay using different cancer cell lines. IC_50_ values of active compounds were determined (Figure 2 and Table 2). Among **1**–**9**, **6** showed the highest activity against all assayed cancer cell lines and was even more active than **2** which is believed to be the most active isohexenylnaphthazarine. Compounds **3** and **4** also exhibited higher cytotoxicity than **2**, while **1**, **5**, **7** and **8** exhibited similar IC_50_ values, with the exception of **7**, which was not active against U251 cells up to 100 µM. Our results indicate that the naphthoquinone moiety is more important for activity than the side chain and that the side chain is a kind of modulator of the activity, an assumption that was also made by Cui *et al*. [24]. Similar observations were also made by Papageorgiou [25] concerning antimicrobial activity. Lu *et al*. [26] reported that also the chiral center does not affect activity. Our findings support this because **1**, which lacks the chiral center, showed comparable activity with the other derivatives and the activities of enantiomers **3** and **9** did not differ significantly.

**Table 2:** IC_50_ values of isolated alkannin derivatives (**1**–**8**), acetylshikonin (**9**) and vinblastine (VBN) against different cancer cell lines. Mean ± SEM, n = 4.

Compound	IC_50_ (µM)			
	CCRF-CEM	MDA-MB-231	U251	HCT 116
**1**	2.31 ± 0.27	13.34 ± 1.35	15.01 ± 1.16	8.39 ± 1.20
**2**	1.67 ± 0.38	27.59 ± 8.25	41.70 ± 5.85	6.53 ± 0.87
**3**	0.98 ± 0.05	6.22 ± 0.46	19.17 ± 1.43	1.52 ± 0.05
**4**	0.53 ± 0.02	5.60 ± 0.36	16.39 ± 0.45	5.64 ± 0.14
**5**	3.32 ± 0.74	12.07 ± 0.13	69.70 ± 17.67	12.50 ± 1.05
**6**	0.39 ± 0.02	4.62 ± 0.14	3.17 ± 0.31	1.75 ± 0.06
**7**	1.22 ± 0.24	30.05 ± 5.92	>100 µM	7.97 ± 0.84
**8**	1.69 ± 0.15	12.21 ± 0.88	25.84 ± 0.66	6.83 ± 0.85
**9**	1.01 ± 0.09	11.33 ± 1.65	15.86 ± 0.67	9.02 ± 0.51
**VBN**	9.4 × 10^−3^ ± 2 × 10^−4^	3.1 × 10^−2^ ± 4.6 × 10^−3^	8.1 × 10^−3^ ± 1.0 × 10^−3^	8.7 × 10^−3^ ± 5.0 × 10^−4^

## 3. Experimental

### 3.1. Chemicals and Reagents

Silica gel 60 and TLC plates Kieselgel 60, 0.2 mm layer thickness, were purchased from Merck Chemical Co. (Darmstadt, Germany). Bands on TLC plates were detected under UV light (254 and 366 nm) and/or after spraying with a 2.5% H_2_SO_4_ and 2.5% vanillin methanolic solutions and heating at 105 °C for 5 min. Dichloromethane was used as an extraction solvent for the compound bands obtained from preparative TLC, using 20 cm × 20 cm plates each one loaded with ≤15 mg of sample. Solvents including *n*-hexane, cyclohexane, ethyl acetate, dichloromethane, diethyl ether and toluene were purchased from Lab Scan Ltd (Dublin, Ireland). BSTFA (*N,O*-bis(trimethylsilyl)trifluoroacetamide) was purchased from Merck (Darmstadt, Germany).

### 3.2. Instruments and Methods

The GC-MS analysis was performed with a Hewlett Packard Gas Chromatograph 5890 Series II Plus linked to a Hewlett Packard 5972 mass spectrometer system equipped with a 30 m long × 0.25 mm i.d. and 0.5 µm film thickness HP5-MS capillary column. The temperature was programmed from 100 to 300 °C at a rate of 4 °C/min. Helium was used as a carrier gas, flow rate 0.7 mL/min. Split ratio 1:20, injector temperature 220 °C, ionization voltage 70 eV. Silylated samples for GC were obtained after addition of excess of BSTFA in pyridine followed by heating at 80 °C for 20 min. ESI-MS spectra were obtained using a 3200 QTRAP LC/MS/MS Applied Biosystems mass spectrometer using an ion spray voltage of +4,500 V, declustering potential of 50 V and entrance potential of 10V. ^1^H- and ^13^C-1D, HSQC, and COSY 2D-NMR spectra were recorded on Bruker Avance 400 and 200 MHz FT-NMR spectrometers. [α]_D_values were measured in dichloromethane using a Perkin Elmer 341 polarimeter.

### 3.3. Plant Callus and Cell Suspension Culture

Callus tissue of *A. euchroma* was maintained on MSA solid medium [5] (50 mL) in 300 mL Erlenmeyer flasks. The medium was supplemented with kinetin (1.0 mg/L) and IAA (0.3 mg/L) prior to autoclaving at 121 °C for 20 min and solidified with 0.8% of DIFCO agar. The cell suspension culture was established by transferring callus tissue into liquid MSA medium and kept on an INFORS AG TR 250 shaker at 105 rpm. Every four weeks 1.5 ± 0.05 g fresh weight of cell aggregates were placed into 250 mL Erlenmeyer flasks containing 50 mL of fresh MSA medium. The callus and cell suspension cultures were cultivated at 25 °C in the dark.

### 3.4. Extraction and Isolation

Lyophilized callus tissue (65.97 g dry weight) and cells from cell suspension culture (47.73 g dry weight) of *A. euchroma* were extracted for 5 days with *n*-hexane in a Soxhlet apparatus. The solvent was evaporated *in vacuo* and the deep red semi-solid residues (0.91 g from the cell suspension culture and 0.59 g from the callus culture) were examined by TLC for their contents. These extracts were subjected to silica gel column chromatography using gradients of cyclohexane/EtOAc (100:0 → 0:100%) and 26 fractions (**A**–**Z**) were collected for the cell suspension extract. Some of these fractions were further subjected to preparative TLC: fraction **A** (207.2 mg) developed with cyclohexane/Et_2_O/AcOH 97/2/1 afforded **1** (11.4 mg) and methyl linolate (2.2 mg); fraction **E** (171.8 mg) developed with cyclohexane/EtOAc/AcOH 90/9/1 afforded **6** (45 mg) as well as a mixture of fatty acid triglycerides (10 mg). 1 mg of the latter was subjected to transesterification/methylation using TMSH (trimethylsulfonium hydroxide) and the obtained fatty acid methyl esters were identified by GC-MS as methyl palmitate (42.8%), methyl stearate (14.7%) and methyl 8-*Z*- or 9-*Z*- or 9-*E*- or 11-*Z*-octadecanoate (42.5%); fractions **G** and **H** (74.4 mg) developed with cyclohexane/Et_2_O/AcOH 87/12/1 afforded **4** (15 mg) and **7** (4.9 mg); **L** and **M** (326 mg) developed with cyclohexane/EtOAc/AcOH 85/14/1 afforded **3** (82.6 mg), linoleic acid (8.0 mg) and β-sitosterol (9.7 mg); fraction **P** (47 mg) developed twice with cyclohexane/EtOAc/AcOH 92/7/1 afforded **10** (6.9 mg). Fractions **S** and **T** (37.3 mg) developed with PhMe/EtOAc/AcOH 95/4/1 afforded **2** (3.8 mg) and **8** (2.6 mg); **U-W** (37.2 mg) developed with cyclohexane/ EtOAc/AcOH 80/19/1 yielded **5** (7.8 mg); finally **Z** (31.8 mg) developed twice with cyclohexane/EtOAc/AcOH 65/34/1 afforded a mixture of 1-monoglycerides (6.1 mg). 1 mg of the latter was subjected to transesterification/methylation using TMSH and the obtained fatty acid methyl esters were identified by GC-MS as methyl palmitate (69.2%), methyl stearate (16.9%) and methyl olate (13.9%). In addition, silylation of a small amount of the 1-monoglyceride mixture lead to the corresponding mixture of the bis(trimethylsilyl) derivatives, which after GC-MS analysis were identified as 2,3-*O*-bis(trimetylsilyl)-1-monoesters of glycerol with palmitic acid (73.8%), oleic acid (8.9%) and stearic acid (17.3%).

Likewise, 20 fractions (**A**–**T**) were collected for the callus culture extract, after the initial silica gel column chromatography using the same elution procedure. Some of these fractions were further subjected to preparative TLC: fraction **A** (152.3 mg) was developed with cyclohexane/Et_2_O/AcOH 97/2/1 and afforded **1** (7.6 mg); **E-H** (141.8 mg) were developed twice with PhMe/AcOH 99/1 and afforded **4** (8.8 mg) and **6** (29.6 mg); finally **K**, **L**, **M**, developed with cyclohexane/EtOAc/AcOH 85/14/1 afforded **9** (41.4 mg) and also GC-MS examination of two other zones revealed the presence of linoleic acid and β-sitosterol.

### 3.5. Compound Characterization

All structures were identified by MS (samples dissolved in methanol) and NMR spectroscopy.

*Deoxyalkannin* (**1**); deep red semi-solid; ^1^H-NMR (400 MHz, CDCl_3_) *δ* 1.60 (3H, *s*, H-6'), 1.70 (3H, *s*, H-5'), 2.31 (2H, *m*, H-2'), 2.64 (2H, *t*, *J* = 7.6 Hz, H-1'), 5.14 (1H, *t*, *J* = 7.2 Hz, H-3'), 6.84 (1H, *s*, H-3), 7.21 (2H, *s*, H-6 and H-7), 12.48 and 12.64 (2 × 1H, 2 × *s*, OH); ^13^C-NMR (50 MHz, CDCl_3_) *δ* 17.8 (C-6'), 25.6 (C-5'), 26.5 (C-2'), 29.7 (C-1'), 111.7 and 111.9 (C-9 and C-10), 122.3 (C-3'), 130.9 and 131.1 (C-6 and C-7), 133.6 (C-4'), 134.5 (C-3), 151.5 (C-2), 162.3 and 162.9 (C-5 and C-8), 183.0 (C-1 and C-4); ESI-MS *m/z* 273 [M+H]^+^.

*Alkannin* (**2**); deep red semi-solid; [*α*]_*D*_^25^: −164 (c = 0.01583); ^1^H-NMR (400 MHz, CDCl_3_) *δ* 1.66 (3H, *s*, H-6'), 1.76 (3H, *s*, H-5'), 2.36 and 2.65 (2 × 1H, 2 × *m*, H-2'), 4.92 (1H, *dd*, *J* = 4.1 and 7.6 Hz, H-1'), 5.21 (1H, *t*, *J* = 7.3 Hz, H-3'), 7.18 (1H, *s*, H-3), 7.21 (2H, *s*, H-6 and H-7), 12.51 and 12.61 (2 × 1H, 2 × *s*, OH); ^13^C-NMR (50 MHz, CDCl_3_) *δ* 18.1 (C-6'), 25.9 (C-5'), 35.7 (C-2'), 68.4 (C-1'), 111.6 and 112.0 (C-9 and C-10), 118.5 (C-3'), 131.9 (C-3), 132.3 and 132.4 (C-6 and C-7), 137.4 (C-4'), 151.4 (C-2), 164.9 and 165.6 (C-5 and C-8), 179.8 and 180.6 (C-1 and C-4); ESI-MS *m/z* 289 [M+H]^+^.

*Acetylalkannin* (**3**); deep red semi-solid; [*α*]_*D*_^25^: −772 (c = 0.00136); ^1^H-NMR (400 MHz, CDCl_3_) *δ* 1.56 (3H, *s*, H-6'), 1.68 (3H, *s*, H-5'), 2.13 (3H, *s*, H-2''), 2.46 and 2.60 (2 × 1H, 2 × *m*, H-2'), 5.11 (1H, *t*, *J* = 7.3 Hz, H-3'), 6.00 (1H, *dd*, *J* = 4.6 and 7.1 Hz, H-1'), 6.97 (1H, *s*, H-3), 7.16 (2H, *s*, H-6 and H-7), 12.40 and 12.56 (2 × 1H, 2 × *s*, OH); ^13^C-NMR (50 MHz, CDCl_3_) *δ* 17.8 (C-6'), 20.8 (C-2''), 25.6 (C-5'), 32.7 (C-2'), 69.4 (C-1'), 111.4 and 111.7 (C-9 and C-10), 117.6 (C-3'), 131.4 (C-3), 132.5 and 132.7 (C-6 and C-7), 135.9 (C-4'), 148.1 (C-2), 166.7 and 167.2 (C-5 and C-8), 169.6 (C-1''), 176.7 and 178.2 (C-1 and C-4); ESI-MS *m/z* 331 [M+H]^+^.

*Isobutyrylalkannin* (**4**); deep red semi-solid; [*α*]_*D*_^25^: −515 (c = 0.00136); ^1^H-NMR (400 MHz, CDCl_3_) *δ* 1.20 (6H, *d*, *J* = 7.1 Hz, H-3'' and H-4''), 1.58 (3H, *s*, H-6'), 1.68 (3H, *s*, H-5'), 2.46 and 2.63 (2 × 1H, 2 × *m*, H-2'), 2.65 (1H, *m*, H-2''), 5.12 (1H, *t*, *J* = 7.3 Hz, H-3'), 6.01 (1H, *dd*, *J* = 4.6 and 7.2 Hz, H-1'), 6.97 (1H, *s*, H-3), 7.18 (2H, *s*, H-6 and H-7), 12.43 and 12.58 (2 × 1H, 2 × *s*, OH); ^13^C-NMR (50 MHz, CDCl_3_) *δ* 17.9 (C-6'), 18.8 and 18.9 (C-3'' and C-4''), 25.7 (C-5'), 32.9 (C-2'), 34.0 (C-2''), 69.0 (C-1'), 111.5 and 111.7 (C-9 and C-10), 117.8 (C-3'), 131.3 (C-3), 132.6 and 132.8 (C-6 and C-7), 136.1 (C-4'), 148.7 (C-2), 166.7 and 166.9 (C-5 and C-8), 175.7 (C-1''), 176.8 and 178.3 (C-1 and C-4); ESI-MS *m/z* 359 [M+H]^+^.

*β-hydroxyisovalerylalkannin* (**5**); deep red semi-solid; [*α*]_*D*_^25^: −147 (c = 0.00136); ^1^H-NMR (400 MHz, CDCl_3_) *δ* 1.30 and 1.31 (2 × 3H, 2 × *s*, H-4'' and H-5''), 1.59 (3H, *s*, H-6'), 1.69 (3H, *s*, H-5'), 2.51 and 2.62 (2 × 1H, 2 × *m*, H-2'), 2.59 (2H, *s*, H-2''), 5.12 (1H, *t*, *J* = 6.7 Hz, H-3'), 6.09 (1H, *dd*, *J* = 4.6 and 7.2 Hz, H-1'), 7.02 (1H, *s*, H-3), 7.18 (2H, *s*, H-6 and H-7), 12.42 and 12.60 (2 × 1H, 2 × *s*, OH); ^13^C-NMR (50 MHz, CDCl_3_) *δ* 17.9 (C-6'), 25.7 (C-5'), 29.1 and 29.2 (C-4'' and C-5''), 32.9 (C-2'), 46.5 (C-2''), 69.1 (C-3''), 69.8 (C-1'), 111.6 and 111.8 (C-9 and C-10), 117.6 (C-3'), 131.3 (C-3), 133.1 and 133.3 (C-6 and C-7), 136.4 (C-4'), 147.5 (C-2), 168.2 and 168.7 (C-5 and C-8), 171.7 (C-1''), 175.3 and 176.9 (C-1 and C-4); ESI-MS *m/z* 389 [M+H]^+^.

*2''-(S)-α-Methylbutyrylalkannin* (**6**); deep red semi-solid; [*α*]_*D*_^25^: −368 (c = 0.00136); ^1^H-NMR (400 MHz, CDCl_3_) *δ* 0.90 (3H, *t*, *J* = 7.5 Hz, H-4''), 1.17 (3H, *d*, *J* = 7.0 Hz, H-5''), 1.49 and 1.69 (2 × 1H, 2 × *m*, H-3''), 1.58 (3H, *s*, H-6'), 1.68 (3H, *s*, H-5'), 2.43 (1H, *m*, H-2''), 2.45 and 2.60 (2 × 1H, 2 × *m*, H-2'), 5.12 (1H, *t*, *J* = 7.5 Hz, H-3'), 6.02 (1H, *dd*, *J* = 4.5 and 7.5 Hz, H-1'), 6.98 (1H, *s*, H-3), 7.16 (2H, *s*, H-6 and H-7), 12.42 and 12.58 (2 × 1H, 2 × *s*, OH); ^13^C-NMR (50 MHz, CDCl_3_) *δ* 11.6 (C-4''), 16.6 (C-5''), 17.9 (C-6'), 25.7 (C-5'), 26.6 (C-3''), 33.0 (C-2'), 41.2 (C-2''), 69.0 (C-1'), 111.6 and 111.8 (C-9 and C-10), 117.8 (C-3'), 131.4 (C-3), 132.7 and 132.8 (C-6 and C-7), 136.0 (C-4'), 148.6 (C-2), 166.8 and 167.3 (C-5 and C-8), 175.3 (C-1''), 176.8 and 178.3 (C-1 and C-4); ESI-MS *m/z* 373 [M+H]^+^.

*Propionylalkannin* (**7**); deep red semi-solid; [*α*]_*D*_^25^: −221 (c = 0.00136); ^1^H-NMR (400 MHz, CDCl_3_) *δ* 1.17 (3H, *t*, *J* = 7.6 Hz, H-3''), 1.58 and 1.69 (2 × 3H, 2 × *s*, H-5' and H-6'), 2.43 (2H, *q*, *J* = 7.6 Hz, H-2''), 2.47 and 2.62 (2 × 1H, 2 × *m*, H-2'), 5.12 (1H, *m*, H-3'), 6.03 (1H, *m*, H-1'), 6.98 (1H, *d*, *J* = 1.0 Hz, H-3), 7.19 (2H, s, H-6 and H-7), 12.44 and 12.60 (2 × 1H, 2 × *s*, OH); ^13^C-NMR (50 MHz, CDCl_3_) *δ* 9.0 (C-3''), 17.9 (C-6'), 25.8 (C-5'), 27.6 (C-2''), 32.8 (C-2'), 69.2 (C-1'), 111.6 and 111.8 (C-9 and C-10), 117.7 (C-3'), 131.4 (C-3), 132.7 and 132.8 (C-6 and C-7), 136.0 (C-4'), 148.4 (C-2), 166.8 and 167.3 (C-5 and C-8), 173.2 (C-1''), 176.8 and 178.3 (C-1 and C-4); ESI-MS *m/z* 345 [M+H]^+^.

*Teracrylalkannin* (**8**); deep red semi-solid; [*α*]_*D*_^25^: −521 (c = 0.00192); ^1^H-NMR (400 MHz, CDCl_3_) *δ* 1.53 and 1.56 (2 × 3H, 2 × *s*, H-6'' and H-7''), 1.57 and 1.68 (2 × 3H, 2 × *s*, H-5' and H-6'), 2.00 (3H, *s*, H-5''), 2.47 and 2.61 (2 × 1H, 2 × *m*, H-2'), 2.94 and 3.02 (2 × 1H, 2 × *d*, *J* = 14.4 Hz, H-2''), 5.12 (1H, *t*, *J* = 7.0 Hz, H-3'), 6.03 (1H, *dd*, *J* = 4.4 and 7.2 Hz, H-1'), 7.01 (1H, *s*, H-3), 7.18 (2H, *s*, H-6 and H-7), 12.42 and 12.59 (2 × 1H, 2 × *s*, OH); ESI-MS, *m/z* 399 [M+H]^+^.

*Acetylshikonin* (**9**); deep red semi-solid; [*α*]_*D*_^25^: + 692 (c = 0.00136), same spectral data as for acetylalkannin (**3**).

*Methyl jasmonate* (**10**); ^1^H-NMR (400 MHz, CDCl_3_) *δ* 0.95 (3H, *t*, *J* = 7.5 Hz), 1.88 (1H, *m*), 2.00–2.45 (10H, *m*), 2.71 (1H, *m*), 3.69 (3H, *s*), 5.25 (1H, *m*), 5.45 (1H, *m*); ^13^C-ΝΜR (50 MHz, CDCl_3_) *δ* 13.3, 20.7, 25.6, 27.3, 37.8, 38.1, 38.9, 51.7, 54.1, 125.1, 134.2, 172.4, 218.8.

### 3.6. Antimicrobial Activity

#### 3.6.1. Microbial Strains

A total of nine microorganisms were assayed among which two Gram-positive bacteria: *Staphylococcus aureus* (ATCC 25923), *Staphylococcus epidermidis* (ATCC 12228) and the four Gram-negative bacteria: *Escherichia coli* (ATCC 25922), *Enterobacter cloacae* (ATCC 13047), *Klebsiella pneumoniae* (ATCC 13883) and *Pseudomonas aeruginosa* (ATCC 227853) as well as the pathogen fungi *Candida albicans* (ATCC 10231), *C. tropicalis* (ATCC 13801) and *C. glabrata* (ATCC 28838).

#### 3.6.2. Antimicrobial Assays

Antimicrobial activity was evaluated using the disc diffusion method by measuring the zone of inhibition. Standard antibiotics netilmicin and 5-flucytocine (Sanofi, Diagnostics Pasteur, Lake Hazeltine Drive Chaska, MT, USA) were used in order to control the sensitivity of the tested bacteria and fungi, respectively. The tested compounds were dissolved in MeOH. For each experiment control disc with pure solvent was used as blind control. All paper discs had a diameter of 6 mm and were deposited on the surface of the seeded trypticase soy agar Petri dishes. The plates were inoculated with the organisms of interest to give a final cell concentration of 10^7^ cells/mL and incubated for 48 h at 37 °C. The fungi were grown on Sabouraud’s agar at 25 °C for 48 h. The experiments were repeated three times and results (diameters in mm) were expressed as mean values [27].

### 3.7. Cell Culture

Human leukemia cells CCRF-CEM and breast cancer cells MDA-MB-231 were cultured in RPMI 1640 medium (Sigma, St. Louis, MO, USA), 2 mM L-glutamine (Sigma), 10% heat-inactivated fetal bovine serum (FBS, PAA Laboratories, Pasching, Austria) and 1% Pen/Strep. (PAA Laboratories). Human glioblastoma cells U251 and colon cancer cells HCT 116 were grown in Dulbecco’s modified Eagle medium (DMEM, Sigma), 2 mM L-glutamine, 10% FBS and 1% Pen/Strep. All cells were kept in a 5% CO_2_ atmosphere at 37 °C. At 90% confluence cells were passaged.

### 3.8. XTT Viability Assay

Cell proliferation kit II (XTT) (Cat. No. 11 465 015 001) was obtained from Roche Diagnostics (Mannheim, Germany). Aliquots (100 µL) of 5 × 10^4^ cells/mL of MDA-MB-231, U251 and HCT 116 cells were seeded in 96-well plates (flat bottom) and grown for 18 h in a humidified 37 °C, 5% CO_2_ atmosphere before substances were added. Control cells were treated with 0.5% DMSO which had no effect on their growth and viability. In case of CCRF-CEM cells, aliquots (100 µL) of 1 × 10^5^ cells/mL were seeded in 96-well plates (flat bottom) and substances were added immediately. All cells were incubated with the substance of interest for 72 h before XTT solution was added. Vinblastine served as a positive control. XTT is a yellow tetrazolium salt (sodium 3'-[1-(phenylaminocarbonyl)-3,4-tetrazolium]-bis(4-methoxy-6-nitro)benzene sulfonic acid hydrate) and cleaved by metabolic active cells into an orange formazan dye. This colour change occurs only in viable cells and can be directly quantified using a scanning multiwell spectrophotometer [28]. Numbers of viable cells were determined with the following formula expressed as percentage of control: (absorbance of treated cells/absorbance of untreated cells) × 100 [29]. IC_50_ values were calculated using SigmaPlot 11.0 (Systat Software Inc., San Jose, CA, USA) and the four parameter logistic curve.

## 4. Conclusions

The *n*-hexane extract from cell suspension culture of *A. euchroma* yielded after several chromatographic separations the compounds deoxyalkannin (**1**), alkannin (**2**), acetylalkannin (**3**), isobutyrylalkannin (**4**), β*-*hydroxyisovalerylalkannin (**5**), 2''-(*S*)-α-methylbutyrylalkannin (**6**), propionylalkannin (**7**) and teracrylalkannin (**8**) along with methyl jasmonate (**10**), methyl linoleate, linoleic acid, β-sitosterol, a triglyceride mixture of palmitic, stearic and 8-*Ζ*- or 9-*Ζ*- or 11-*Ζ *or 9-*Ε*-octadecenoic acid and also a mixture of 1-monoglycerides of palmitic, stearic and oleic acid. Likewise from the *n*-hexane extract of callus culture of *A. euchroma* the isohexenylnaphthazarins deoxyalkannin (**1**), isobutyrylalkannin (**4**), 2''-(*S*)-α-methylbutyrylalkannin (**6**) and acetylshikonin (**9**) were obtained after several separations. In addition linoleic acid and β-sitosterol were identified after GC-MS analysis of some fractions. Among them **4** and **9** are novel for the *Arnebia* genus while naphthoquinones **2**–**8** are isolated from *A. euchroma in vitro* cultures for the first time. Furthermore **10** and the 1-monoglycerides of palmitic, stearic and oleic acid are probably reported for the first time within Boraginaceae family. As regards the tested antimicrobial activity of **1**–**9**, **2**, **4**, **5** and **9** where the most active against all the assayed microorganisms while acetylshikonin (**9**) was found much more active than its enantiomer acetylalkannin (**3**). Compound **6** exhibited the most potent cytotoxic activity against the tested cancer cell lines while it was estimated that the structure of the side chain or its chirality have a minor effect on their cytotoxic activity.
